# Supplementary material for: Effects of Acid-Anhydride-Modified Cellulose Nanofiber on Poly(Lactic Acid) Composite Films
Source: Nanomaterials (Basel). 2021 Mar 17;11(3):753. doi: 10.3390/nano11030753 (PMC8002836; doi:10.3390/nano11030753)
Supplement: Supplementary file 1 [file nanomaterials-11-00753-s001.pdf]

Supplementary Materials

Effects of Acid-Anhydride-Modified Cellulose Nanofiber on Poly(Lactic Acid) Composite Films

Naharullah Jamaluddin, Yu-I Hsu *, Taka-Aki Asoh and Hiroshi Uyama *

Department of Applied Chemistry, Graduate School of Engineering, Osaka University, 2-1 Yamadaoka, Suita, Osaka 565-0871, Japan; naharullah@chem.eng.osaka-u.ac.jp (N.J.); asoh@chem.eng.osaka-u.ac.jp (T.-A.A.)

* Correspondence: yuihsu@chem.eng.osaka-u.ac.jp (Y.-I.H.); uyama@chem.eng.osaka-u.ac.jp (H.U.); Tel.: +81-6-6879-7364 (Y.-I.H.); (H.U.), Fax: +81-6-6879-7367 (Y.-I.H.); (H.U.)

Derivation of the equations from EDX spectroscopy

1. CNF = $C_6H_{10}O_5$ (M_w = 162 g/mol)

   Mass percentages (excluding H): C = 47.3%, O = 52.7%

   Theoretical ratio of C:O $= \frac{\mathrm{C}}{\mathrm{O}} = \frac{47.3}{52.7} = 0.897$

2. Assuming that 100% CNF is modified to CNFa,

   CNFa = $C_{12}H_{16}O_8$ (M_w = 288 g/mol)

   Mass percentages (excluding H): C = 52.9%, O = 47.1%

   $A\% = \text{percentage of CNF acetylate (CNFa)}$

   $B = \text{ratio of C: O} = \frac{\mathrm{C}}{\mathrm{O}}$

   $B = \frac{\mathrm{C}}{\mathrm{O}} = \frac{Mw \text{ of single C [No of C acetylate } (A\%) + \text{No of C in CNF } (100\% - A\%)]}{Mw \text{ of single O [No of O acetylate } (A\%) + \text{No of O in CNF } (100\% - A\%)]}$

   $B = \frac{\mathrm{C}}{\mathrm{O}} = \frac{12[12(A\%)+6(100\%-A\%)]}{16[8(A\%)+5(100\%-A\%)]}$

   Simplify A%:

   $A\% = \frac{900-1000B}{6B-9}$ (**S1**)

3. Assuming that 100% CNF is modified to CNFp,

   CNFp = $C_{15}H_{22}O_8$ (M_w = 330 g/mol)

   Mass percentages (excluding H): C = 58.4%, O = 41.6%

   $P\% = \text{percentage of CNF propionate (CNFp)}$

   $B = \text{ratio of C: O} = \frac{\text{C}}{\text{O}}$

   $$B = \frac{\text{C}}{\text{O}} = \frac{Mw \text{ of single C [No of C propionate } (P\%) + \text{No of C in CNF } (100\% - P\%)]}{Mw \text{ of single O [No of O propionate } (P\%) + \text{No of O in CNF } (100\% - P\%)]}$$

   $$B = \frac{\text{C}}{\text{O}} = \frac{12[15(P\%) + 6(100\% - P\%)]}{16[8(P\%) + 5(100\% - P\%)]}$$

   Simplify P%:

   $$P\% = \frac{200\ (10B - 9)}{3\ (9 - 4B)} \ (\textbf{S2})$$

4. Assuming that 100% CNF is modified to CNFb,

   CNFb = $C_{18}H_{28}O_8$ (M_w = 372 g/mol)

   Mass percentages (excluding H): C = 62.8%, O = 37.2%

   $B\% = \text{percentage of CNF butyrate (CNFb)}$

   $B = \text{ratio of C: O} = \frac{\text{C}}{\text{O}}$

   $$B = \frac{\text{C}}{\text{O}} = \frac{Mw \text{ of single C [No of C butyrate } (B\%) + \text{No of C in CNF } (100\% - B\%)]}{Mw \text{ of single O [No of O butyrate } (B\%) + \text{No of O in CNF } (100\% - B\%)]}$$

   $$B = \frac{\text{C}}{\text{O}} = \frac{12[18(B\%) + 6(100\% - B\%)]}{16[8(B\%) + 5(100\% - B\%)]}$$

   Simplify B%:

   $$B\% = \frac{450 - 500B}{3\ (B - 3)} \ (\textbf{S3})$$

5. $\text{Degree of substitution (DS)} = \frac{\text{percentage modified}}{100\%} \times 3$ (**S4**)

From the EDX instrument, the value of B can be obtained, which is the C:O mass ratio. Therefore, the DS of m-CNF can be calculated from the derived equations. The full details of the results from EDX analysis, including the CNF, are given in **Table S1**.

Table S1. Details of the mass concentrations obtained from EDX analysis.

Species	Mass concentration (%)		Ratio of C:O	Percent modified (%)	Degree substitutions (DS)
	C	O			
CNF	47.7	52.3	0.91	0	0
CNFa1	48.6	51.4	0.95	13.7	0.41
CNFa2	49.5	50.5	0.98	25.0	0.75
CNFa4	49.7	50.3	0.99	29.4	0.88
CNFp1	50.3	49.7	1.01	15.1	0.45
CNFp2	52.1	47.9	1.09	26.9	0.81
CNFp4	52.7	47.3	1.11	31.4	0.94
CNFb1	50.4	49.6	1.02	9.8	0.29
CNFb2	51.9	48.1	1.08	15.5	0.47
CNFb4	53.9	46.1	1.17	24.5	0.74

Morphology of m-CNF from SEM images.

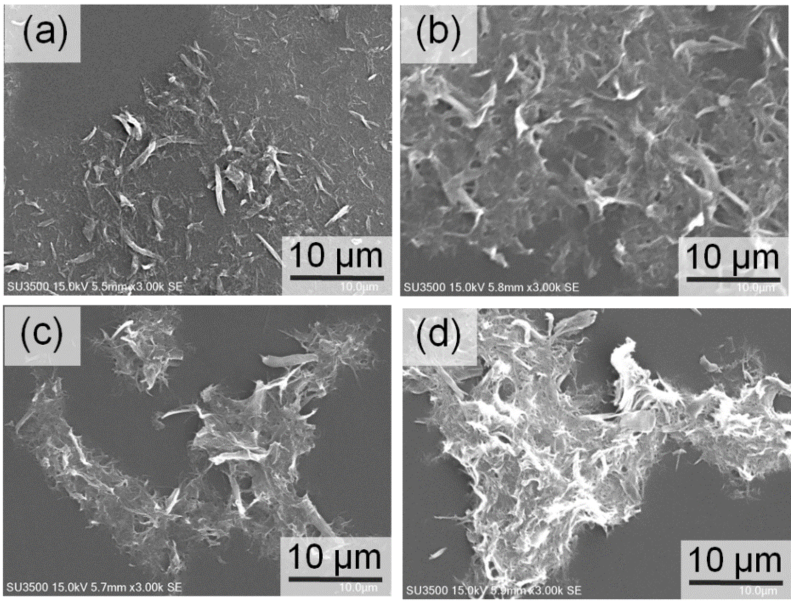

Figure S1. SEM images of (a) CNF, (b) CNFa4 (DS = 0.88), (c) CNFp4 (DS = 0.94), and (d) CNFb4 (DS = 0.74) at ×3000 magnification.

Mechanical properties of PLA/m-CNF composite films from stress-strain curves.

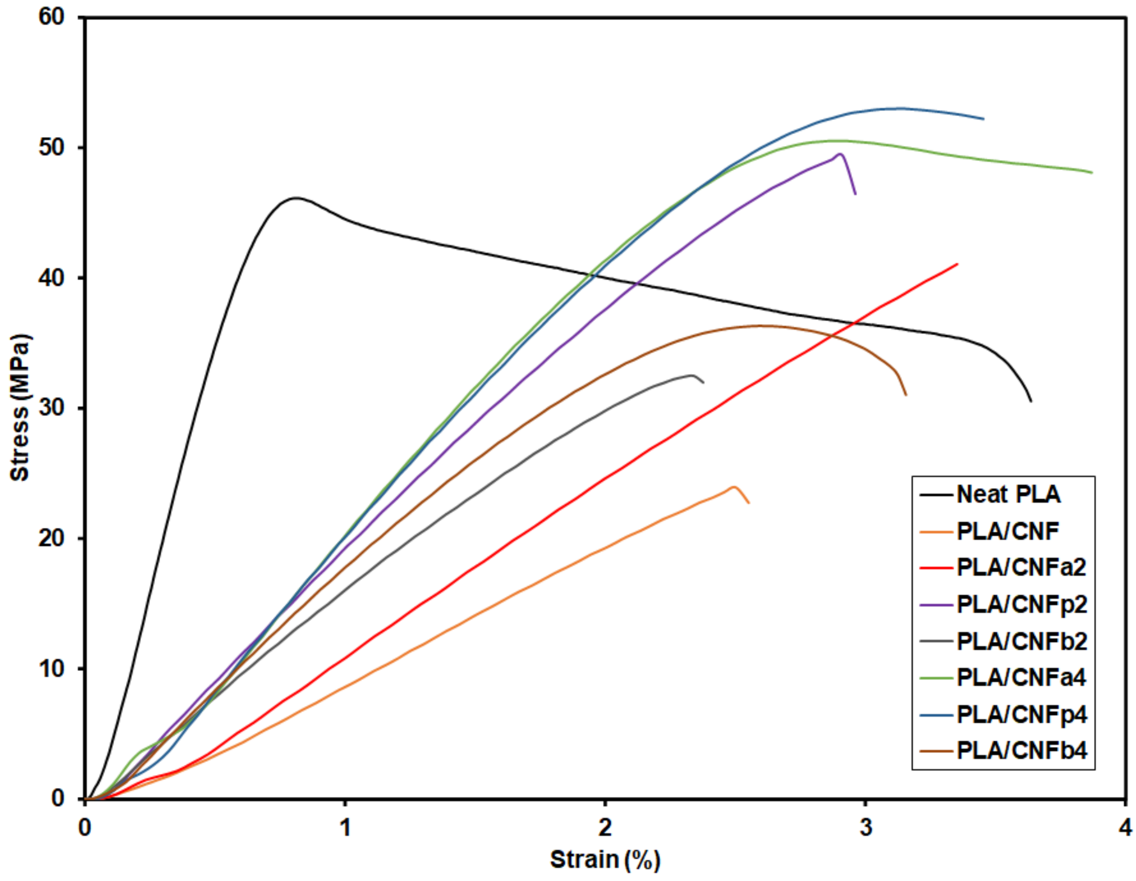

Figure S2. Stress-strain curves of PLA/m-CNF composite films.

Thermal properties of PLA/m-CNF composite films for the degree of crystallinity.

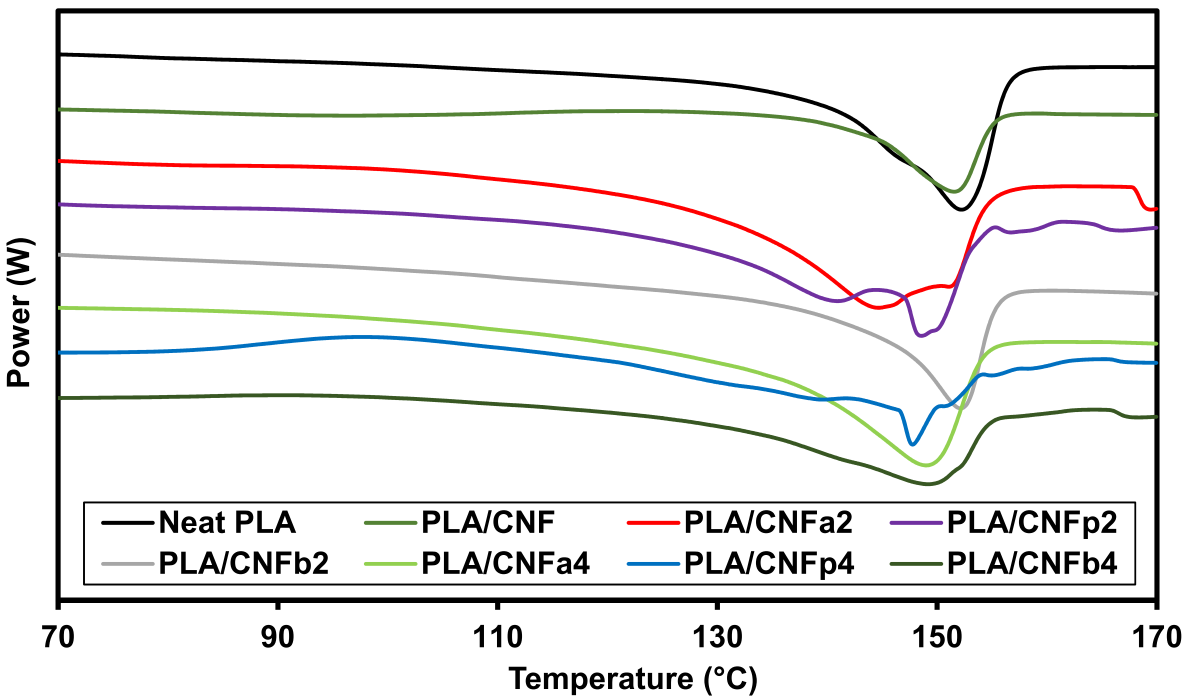

Figure S3. DSC thermograms of PLA/m-CNF composite films.
